# Severe loss of mechanical efficiency in COVID‐19 patients

**DOI:** 10.1002/jcsm.12739

**Published:** 2021-06-08

**Authors:** Eulogio Pleguezuelos, Amin Del Carmen, Gemma Llorensi, Jessica Carcole, Paula Casarramona, Eva Moreno, Pilar Ortega, Mateo Serra‐Prat, Elisabet Palomera, Marc M. Miravitlles, Joan Carles Yebenes, Ramón Boixeda, Lluis Campins, Koldo Villelabeitia‐Jaureguizar, Manuel Vicente Garnacho‐Castaño

**Affiliations:** ^1^ Physical Medicine and Rehabilitation Department Hospital de Mataró Mataró Spain; ^2^ Department of Experimental Science and Healthcare, Faculty of Health Sciences Universitat Pompeu Fabra Barcelona Spain; ^3^ Physical Medicine and Rehabilitation Department Hospitalet General Hospital L'Hospitalet de Llobregat Spain; ^4^ Pneumology Department Hospital de Mataró Mataró Spain; ^5^ Research Unit Consorci Sanitari del Maresme Mataró Spain; ^6^ Pneumology Department Hospital Universitari Vall d'Hebron, Vall d'Hebron Institut de Recerca (VHIR), Vall d'Hebron Barcelona Hospital Campus, CIBER de Enfermedades Respiratorias (CIBERES) Barcelona Spain; ^7^ Critical Care Department Hospital de Mataró Mataró Spain; ^8^ Department of Internal Medicine Hospital de Mataró, CSDM Mataró Spain; ^9^ Grup d'Estudi al Maresme de la Pneumònia Adquirida en la Comunitat i la MPOC (GEMP@C) Mataró Spain; ^10^ Department of Pharmacy Hospital de Mataró, CSdM Mataró Spain; ^11^ Physical Medicine and Rehabilitation Department Infanta Elena University Hospital Valdemoro Spain; ^12^ GRI‐AFIRS, School of Health Sciences, TecnoCampus Universitat Pompeu Fabra Mataró Spain

**Keywords:** SARS‐CoV‐2, Cardiopulmonary exercise test, COPD, Ischaemic heart disease, Muscular dysfunction

## Abstract

**Background:**

There is limited information about the impact of coronavirus disease (COVID‐19) on the muscular dysfunction, despite the generalized weakness and fatigue that patients report after overcoming the acute phase of the infection. This study aimed to detect impaired muscle efficiency by evaluating delta efficiency (DE) in patients with COVID‐19 compared with subjects with chronic obstructive pulmonary disease (COPD), ischaemic heart disease (IHD), and control group (CG).

**Methods:**

A total of 60 participants were assigned to four experimental groups: COVID‐19, COPD, IHD, and CG (*n* = 15 each group). Incremental exercise tests in a cycle ergometer were performed to obtain peak oxygen uptake (VO_2_peak). DE was obtained from the end of the first workload to the power output where the respiratory exchange ratio was 1.

**Results:**

A lower DE was detected in patients with COVID‐19 and COPD compared with those in CG (*P* ≤ 0.033). However, no significant differences were observed among the experimental groups with diseases (*P* > 0.05). Lower VO_2_peak, peak ventilation, peak power output, and total exercise time were observed in the groups with diseases than in the CG (*P* < 0.05). A higher VO_2_, ventilation, and power output were detected in the CG compared with those in the groups with diseases at the first and second ventilatory threshold (*P* < 0.05). A higher power output was detected in the IHD group compared with those in the COVID‐19 and COPD groups (*P* < 0.05) at the first and second ventilatory thresholds and when the respiratory exchange ratio was 1. A significant correlation (*P* < 0.001) was found between the VO_2_peak and DE and between the peak power output and DE (*P* < 0.001).

**Conclusions:**

Patients with COVID‐19 showed marked mechanical inefficiency similar to that observed in COPD and IHD patients. Patients with COVID‐19 and COPD showed a significant decrease in power output compared to IHD during pedalling despite having similar response in VO_2_ at each intensity. Resistance training should be considered during the early phase of rehabilitation.

## Introduction

Coronavirus disease 2019 (COVID‐19) is caused by severe acute respiratory syndrome coronavirus type 2 (SARS‐CoV‐2) and was first identified in Wuhan, Hubei, China, in December 2019.^1^ The disease spread very rapidly to the rest of China and later worldwide. A state of alarm was declared in Spain on 16 March 2020 with the aim of managing the emergency health care situation produced by COVID‐19. Up to 6 November 2020, a total of 1 328 832 confirmed cases and 38 883 deaths by COVID‐19 have been reported in Spain.^2^

As previously demonstrated by other coronaviruses,^3^ SARS‐CoV‐2 can infect different systems that share the same ACE‐2 receptors present in the respiratory system. Therefore, most of the extrapulmonary manifestations occur in the organs or systems with cells that express ACE‐2 receptors (heart, central nervous system, and muscle, among others).^4^ The inflammatory response induced in the airway by SARS‐CoV‐2 can also lead to multisystemic inflammation that can affect almost all organ systems, including the musculoskeletal system.^1^, ^5^ It has been described that the musculoskeletal system is seriously burdened in patients with moderate to severe SARS infection by causing significant skeletal muscle, bone, joint, and neurological disorders.^6^, ^7^ However, there is limited information about the impact of COVID‐19 on the muscular system, despite the generalized weakness and fatigue that patients report after overcoming the acute phase of the infection.

Mechanical efficiency refers to the ability of an individual to transfer energy consumed into external work. In other words, poorer efficiency will increase the percentage of maximal oxygen uptake (VO_2_max) required to sustain a given mechanical work. Reduced mechanical efficiency indicates that more energy is consumed at a given work output, increasing the adenosine triphosphate (ATP) cost of contraction (ATP consumed per work output).^8^

Several slightly varying indicators have been proposed for the assessment of mechanical efficiency. Concretely, delta efficiency (DE) is defined as the relationship between the change in external work (Δ*W*_ext_) and the change in total energy expenditure (Δ*E*_tot_).^9^ DE is considered a valid and predictive parameter of the musculoskeletal efficiency in cycling.^9^, ^10^ This is also due, at least partly, to the fact that the effect of various metabolic processes not contributing to work performance is removed. In this regard, the work of stabilizing muscles, and the work cost of respiratory muscles,^11^ the movement cost of the lower limbs of the body,^12^ and the basal metabolic rate,^9^ are not considered during DE assessment. Thus, DE may be more effective for understanding the efficiency of the musculoskeletal system. The performance of patients with impaired muscle efficiency decreases and, therefore, they may be limited in terms of physical activity.^13^ Consequently, analysis of mechanical efficiency could be valuable for the detection of muscle dysfunction and the evaluation of any subsequent adaptation in response to exercise.^14^

Taking into consideration that COVID‐19 patients may present an important muscle dysfunction, this study aimed to detect impaired muscle efficiency by evaluating DE in COVID‐19 patients compared with subjects with chronic obstructive pulmonary disease (COPD), ischaemic heart disease (IHD), and healthy controls.

## Methods

This was a cross‐sectional, observational study aiming to evaluate DE in COVID‐19 patients. All patients provided informed consent to participate in the study.

The Ethics and Research Committee of the Mataró Hospital approved the study (Codi CEIm: 89/20). The protocol was conducted in accordance with the principles of the Declaration of Helsinki, Good Clinical Practice, and the applicable and local regulatory requirements.

### Participants

The study participants included adult patients who required admission to the intensive care unit (ICU) for presenting respiratory distress syndrome secondary to bilateral COVID‐19 pneumonia. Healthy volunteers (control group) and individuals with COPD or IHD were also recruited from existing databases or the outpatient Cardiac and Pulmonary Rehabilitation Unit of the *Hospital de Mataró* (*Consorci Sanitiari del Maresme*) (*n* = 15 each experimental group).

The adjusted morbidity groups were obtained for all the study participants. The adjusted morbidity group groups morbidities according to data of patient diagnoses encoded in the primary care and hospital medical care histories adjusted for the encoding date (acute or chronic processes).^15^, ^16^

To be eligible, individuals with COPD had to have a post‐bronchodilator spirometry test showing a forced expiratory volume in the first second/forced vital capacity (FEV1/FVC) <0.7 and FEV1 (% predicted) <70%. Individuals with IHD had to have angiographic evidence of disease. Patients with COPD or IHD had been clinically stable for 6 months, without any deterioration in symptoms or episodes of angina in IHD patients. Medications were taken as recommended by the participants' physicians during the study.

For COVID‐19 patients, data related to admission were collected, and the Acute Physiologic and Chronic Health Evaluation (APACHE) II was determined as a predictive system for disease severity and prognosis in patients in the ICU.^17^ The assessment of patients with COVID‐19 was carried out 8 weeks after discharge from hospital.

The exclusion criteria for the four cohorts were severe neurological disease, active oncological disease, joint problems preventing the cardiopulmonary exercise test (CPET), or inability to understand or comprehend the guidelines for performing the CPET.

### Cardiopulmonary exercise test

The CPET was performed on an electro‐mechanically braked bicycle ergometer (Ergoline900S, Ergoline GmbH, Bitz, Germany). The cycling position, which is known to affect energy expenditure, was standardized by adopting a top bar position. Saddle height was adjusted according to the participant's leg length, and knee flexion was between 20° or 30°. Toe‐clips were used, and the participants were instructed to stay seated during the test. The subjects were required to maintain a constant pedal cadence between 50 and 70 revolutions per minute.

An individualized exercise protocol was performed in all patients and was tailored to each patient's physical condition, with gradual increments of 5, 10, 15, or 20 W·min^−1^. The required exercise time was between 6 and 12 min in order to respect the proper kinetics of oxygen consumption (VO_2_) and maintain a linear relationship between VO_2_, exercise workload and heart rate during CPET. Throughout the test, the patients were kept under continuous 12‐lead electrocardiographic‐monitoring, and blood pressure was established every 3 min.

VO_2_ was determined breath by breath using an automated system (Ultima CardiO2, Medical Graphics Corporation, St. Paul, MN, USA). Calibration was performed prior to each test using standard gases of known oxygen and carbon dioxide concentrations as well as a calibration syringe.

The first and second ventilatory thresholds (VT1 and VT2) were determined following the method of ventilatory equivalents (VE·VO_2_^−1^ and VE·VCO_2_^−1^) described by Skinner *et al*.^18^ VT1 corresponds to an increment of the VE·VO_2_^−1^ ratio without an increased VE·VCO_2_^−1^ ratio, and with an increased concentration of oxygen fraction (PetO_2_). VT2 corresponds to an increment of the VE·VCO_2_^−1^ ratio and a fractional decrease in the concentration of CO_2_ (PetCO_2_).

### Outcomes

Mechanical efficiency was calculated as the ratio of work accomplished per minute (Watts converted to kcal·min^−1^) and the energy expended per minute (kcal·min^−1^). The conversion factor 69.7 W·kcal^−1^·min^−1^ was used for estimation of the work accomplished.^19^ An equation based on the thermal equivalent of oxygen for the non‐protein respiratory quotient was used to estimate the energy expended^20^: Energy expended (kcal·min^−1^) = VO_2_·(1.2341·RER + 3.8124).

The mean values measured during the last 30 s of each workload were considered for this estimation.^19^ Finally, DE was calculated as the inverse of the slope in the linear regression (*y* = *ax* + *b*), where *y* is the rate of expended energy (kcal·min^−1^) and *x* is the rate of accomplished work (kcal·min^−1^).^9^ This value was obtained from the end of the first workload, depending on the physical condition of the participants, until the power output where the respiratory exchange ratio (RER) was 1.^19^, ^21^

Secondary outcomes included the total exercise time, VO_2_peak (mL·kg^−1^·min^−1^),VE (L·min^−1^), peak power (W), VO_2_ at VT1 (mL·kg^−1^·min^−1^), VE at VT1 (L·min^−1^), power at VT1 (W), VO_2_ at VT2 (mL·kg^−1^·min^−1^), VE at VT_2_ (L·min^−1^) and power at VT2 (W).

### Statistical analysis

The Shapiro–Wilk test was used to check the normal distribution of the data, which are reported as mean and standard deviation (SD), mean, and confidence intervals (95% CI). To compare the differences between the four experimental groups (healthy control group, COVID‐19, COPD, and IHD), a univariate general linear model was applied. Bonferroni adjustment was used to identify multiple comparisons among experimental groups.

The magnitude of the response to both experimental conditions was estimated by partial eta‐squared (*η*_p_^2^). The scale for classification of *η*_p_^2^ was 0.10 = small, 0.25 = medium, and 0.40 = large.^22^ Statistical power was also calculated.

Total exercise time, VO_2_, VE, and power output during CPET were compared by one‐way analysis of variance (ANOVA). When significant differences emerged, Bonferroni's post hoc was applied to establish differences between experimental groups.

Significance was set at *P* < 0.05. All statistical procedures were applied using the software package SPSS version 25.0 for Mac (SPSS Inc., Chicago, IL, USA).

## Results

### Patients

*Table* 1 describes the characteristics of the groups. Differences were observed in respiratory function tests among the four study groups, except between the COVID‐19 and IHD groups (FVC, L, *P* = 0.361; FVC%, *P* = 0.840; FEV1, L, *P* = 0.805; FEV1, %, *P* = 0.676; FEV1/FVC%, *P* = 0.086). Likewise, and given the study pathologies, we observed statistically significant differences in morbidity among the four study groups (*Table* 1). *Table* 2 describes the most relevant clinical characteristics of the COVID‐19 patients during hospitalization.

**Table 1:** Patients' characteristics

	HG	COVIDG	COPDG	HDG	*P* value
Age (years)	52.2 (4.9)	54.6 (9.1)	56.9 (7.1)	54.4 (8.5)	0.369
Men (%)	100	100	100	100	—
Body mass Index (kg/m^2^)	24.2 (3.5)	29.1 (4.4)	28.3 (6.51)	28.2 (4.0)	0.552
Adjusted morbidity groups (%)	—	—	—	—	<0.001
Basal risk	100%	33.3%	0%	0%	—
Low risk	0%	46.6%	13.3%	6.6%	—
Moderate risk	0%	13.4%	53.3%	46.7%	—
High risk	0%	6.7%	26.7%	46.7%	—
Very high risk	0%	0%	6.7%	0%	—
FVC (L)	5.1 (1.1)	3.8 (1.1)	2.7 (0.7)	4.1 (0.8)	0.007
FVC (%)	103.2 (15.8)	87.3 (17.1)	68.3 (15.2)	87.2 (9.7)	0.002
FEV1 (L)	3.9 (0.8)	3.1 (0.8)	1.4 (0.6)	3.2 (0.8)	<0.001
FEV1 (%)	102.2 (17.7)	88.1 (24.1)	48.1 (21.1)	87.9 (15.1)	<0.001
FEV1/FVC, %	79.9 (7.4)	82 (6.3)	53.3 (16.6)	77.2 (9.1)	<0.001

COPDG, chronic obstructive pulmonary disease group; COVIDG, COVID‐19 group; FEV1, forced expiratory volume in the first second; FVC, forced vital capacity; HDG, heart disease group; HG, healthy group; SD, standard deviation.

**Table 2:** Patients' characteristics COVID‐19

ICU admission days*	11.6 (5.8)
Days of hospital admission*	23.2 (3.7)
Days mechanical ventilation*	10.1 (5.1)
Tracheostomy (no *n*, %)	100%
Prone positioning (yes, %)	86.6%
APACHE II*	11.6 (4)
Pa/Fi 24 h* post VM	168.4 (87.4)
D‐dimer ICU admission*	673.6 (457.8)
PCR admission*	16.9 (12.4)
Lymphocytes admission*	687.3 (212.4)
Azithromycin (yes, %)	100%
Hydroxychloroquine (yes, %)	100%
Lopinavir (yes, %)	100%
Tocilizumab (yes, %)	53.3%
Interferon beta 1β (yes, %)	40%
Corticosteroid bolus (yes, %)	80%

APACHE, Acute Physiologic and Chronic Health Evaluation; ICU, intensive care unit.

* Data are provided as mean and standard deviation (SD).

### Delta efficiency

**Figure 1:**
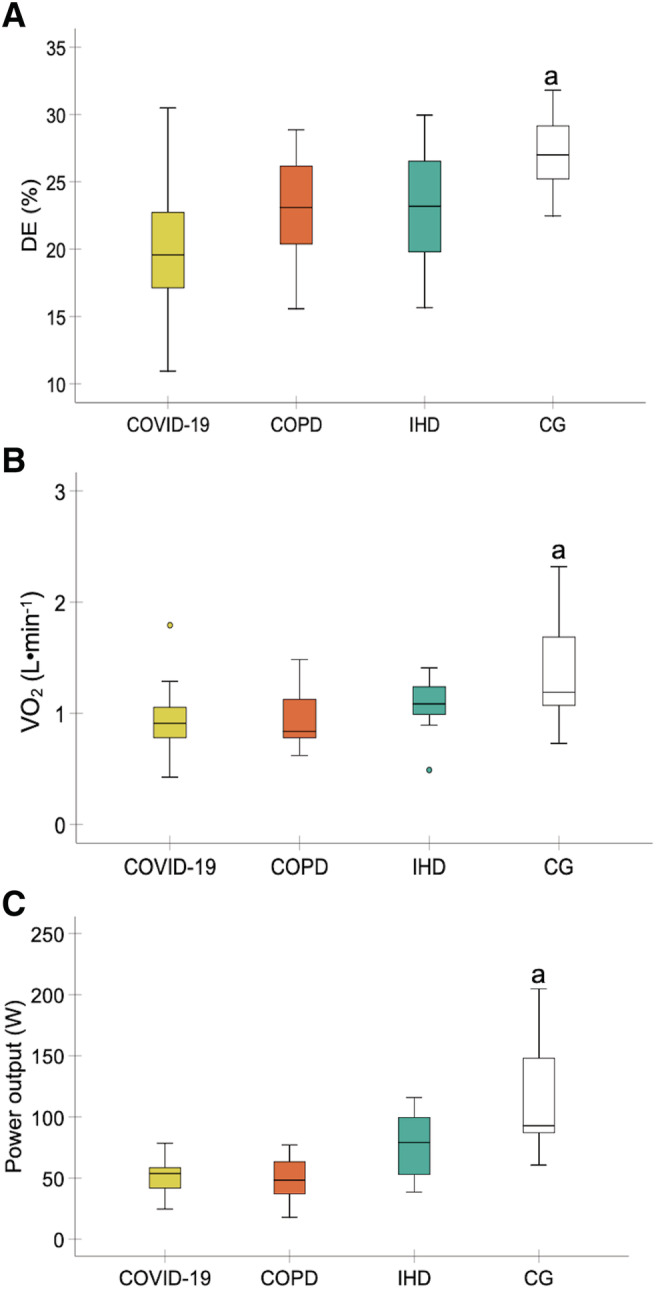
Comparisons in delta efficiency (*A*), VO_2_ when the RER was 1 (*B*), and peak power when the RER was 1 (*C*) between the experimental groups. Abbreviations: CG, healthy control group; COPD, chronic obstructive pulmonary disease; DE, delta efficiency; IHD, ischaemic heart disease; RER, respiratory exchange ratio; VO_2_, oxygen uptake. ^a^Higher DE, VO_2_, and peak power were detected in the healthy control group compared with the COVID‐19 and COPD groups (*P* < 0.05). (*n* = 15 each experimental group).

There were significant differences between experimental groups in DE (*F* = 7.92; *P* < 0.001, *η*_p_^2^ = 0.30, SP = 0.99). Bonferroni's multiple comparisons showed a greater DE in the healthy control group than in the COVID‐19 and COPD groups (*P* < 0.001 and *P* = 0.033, respectively). No significant differences were found between the control group and the IHD group (*P* = 0.052). No significant differences were detected between the pathologies (*P* > 0.05) (*Figure* 1).

### Secondary outcomes

To determine DE, VO_2_, and power output were obtained where the RER was 1. VO_2_ and power output were lower in patients with COVID‐19 and COPD compared with the control group (*P* < 0.01). Power output was higher in patients with IHD compared with patients con COVID‐19 and COPD (*P* < 0.05). No significant differences were detected between patients with COVID‐19 and COPD in both variables (*P* > 0.05). No significant differences were found between patients with IHD and control group in both variables (*P* > 0.05) (*Figure* 1).

The data corresponding to VO_2_, VE, power output, and total test time during CPET at VT1, VT2, and peak intensity are presented in *Table* 3. VO_2_, VE, power output, and total exercise time were lower in the three groups with diseases than in the healthy group (*P* < 0.05) at VT1, VT2, and peak intensities. When comparing the study groups with pathologies, a higher peak power was found in IHD than in COPD patients (*P* < 0.05). Power output was greater in the IHD than in the COVID‐19 and COPD groups (*P* < 0.05) at VT1 and VT2. No other significant differences were detected among the experimental groups (*P* > 0.05).

**Table 3:** Differences in total exercise time, VO_2_, VE, and power output at VT1 and VT2, and peak intensities between experimental groups

	HG	COVIDG	COPDG	IHDG	*P* value
Total exercise time (min:s)	11:20^a^ (10:14–12:25)	8:14 (7:01–9:28)	7:22 (6:25–8:20)	9:04 (7:36–10:32)	<0.001
VO_2_peak (mL·kg^−1^·min^−1^)	32.31^a^ (28.32–36.31)	17.30 (14.82–19.78)	14.35 (12.97–15.73)	18.82 (15.64–22)	<0.001
VE (L·min^−1^)	83.35^a^ (67.33–99.36)	55.05 (45.94–64.15)	38.19 (33.11–43.28)	58.79^c^ (49.09–68.50)	<0.001
Peak power (W)	215.60^a^ (181.84–249.37)	89.67 (69.74–109.60)	74.67 (62.79–86.54)	130.93^c^ (102.25–159.62)	<0.001
VO_2_ at VT1 (mL·kg^−1^·min^−1^)	14.40^a^ (12.33–16.47)	8.94 (7.89–9.99)	9.25 (8.19–10.30)	10.58 (9.03–12.13)	<0.001
VE at VT1 (L·min^−1^)	24.75 (19.81–29.70)	21.42 (17.61–25.19)	21.57 (18.73–24.42)	24.54 (20.84–28.24)	0.394
Power at VT1 (W)	84.47^a^ (68.74–100.19)	29.67 (17.51–41.83)	28.20 (15.39–41.01)	61.60^b^ (45.73–77.47)	<0.001
VO_2_ at VT2 (mL·kg^−1^·min^−1^)	25.18^a^ (21.78–28.58)	12.80 (11.34–14.26)	12.14 (10.56–13.72)	14.83 (12.53–17.13)	<0.001
VE at VT2 (L·min^−1^)	53.21^a^ (42.02–64.41)	35.81 (29.71–41.92)	29.92 (25.11–34.72)	39.08 (33.11–45.04)	<0.001
Power at VT2 (W)	176.53^a^ (147.48–205.58)	69.20 (52.53–85.87)	62.21 (49.36–75.04)	108.41^b^ (87.11–129.69)	<0.001

COPDG, chronic obstructive pulmonary disease group; COVIDG, COVID‐19 group; HG, healthy group; IHDG, ischaemic heart disease group; VE, minute ventilation; VO_2_, oxygen uptake; VT1, first ventilatory threshold; VT2, second ventilatory threshold.

Data are provided as mean and 95% confidence intervals (95% CIs).

^a^ Significantly different from COVIDG, COPDG, and IHDG (*P* < 0.05).

^b^ Significantly different from COVIDG and COPDG at VT1 and VT2 intensities (*P* < 0.05).

^c^ Significantly different from COPDG (*P* < 0.05).

**Figure 2:**
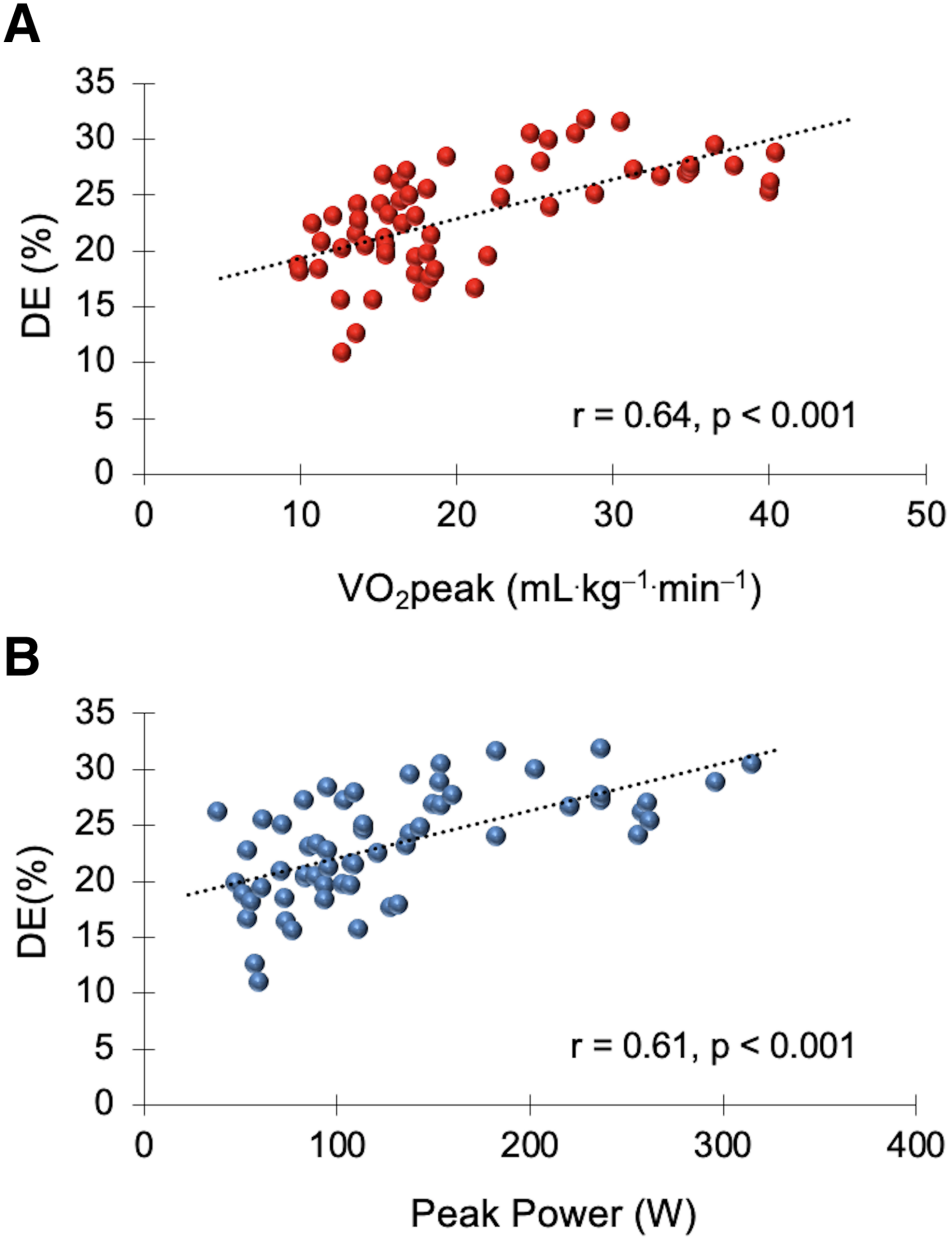
Correlations between delta efficiency (DE) and peak oxygen uptake (VO_2_peak) (*r* = 0.64, *P* < 0.001) (*A*); peak power output in watts (W) (*r* = 0.61, *P* < 0.001) (*B*) (all participants, *n* = 60).

A significant correlation (*r* = 64, *P* < 0.001) was found between the VO_2_peak and DE and between the peak power output and DE (*r* = 61, *P* < 0.001) (*Figure* 2). No other correlations were detected (*P* > 0.05).

## Discussion

To our knowledge, this is the first study to examine mechanical efficiency during exercise in patients who have had COVID‐19 infection and required admission to the ICU due to adult respiratory distress. The most relevant finding was that patients with COVID‐19 and COPD presented reduced DE compared with healthy adult volunteers (control group). It was noteworthy that DE did not change among the experimental groups with cardiorespiratory diseases. Furthermore, no differences were observed between patients with IHD and the control group.

Other relevant findings showed a lower total exercise time, VO_2_, VE (not at VT1), and power output in the three disease groups compared with the control group after CPET. When the three pathologies were compared, the power output in patients with COVID‐19 and COPD was lower than that of patients with IHD at intensities of VT1 and VT2 and when the RER was 1.

Unfortunately, at present, the DE results of our study cannot be reinforced by previous studies in patients with COVID‐19 and, furthermore, research comparing patients with COPD and IHD are limited. The DE observed in patients with COVID‐19 (19.9%) were slightly lower (no significant) than those obtained in patients with COPD and IHD (~23%), and significantly lower compared to the control group (~27%). Other studies have reported mean DE values of ~25–26% in patients with COPD and with heart failure with reduced ejection fraction during submaximal cycling.^23^, ^24^ Perrault *et al*. did not find significant differences between patients with COPD (26.3%) and a control group (24.8%).^25^ According to a review article,^26^ the mean DE of 14 studies was 23.8 ± 2.6%. Similar DE (23.8%) was reported in another study with sport science students.^27^ Given the reported results in these studies, it appears that COVID‐19 patients may have decreased mechanical efficiency compared with healthy people and about 20% could be a reference value for DE. More studies with COVID‐19 patients are needed to corroborate such claims.

Several clinical sequelae should be considered to understand a decrease in mechanical efficiency in patients with COVID‐19. Survivors of SARS‐CoV‐2 are highly prone to developing severe clinical respiratory, cardiovascular, and psychosocial sequelae. These sequelae are linked to important physical decline and significant fatigue.^28^ In addition, muscle soreness, muscle fatigue, and weakness are stated symptoms in COVID‐19 patients.^29^

Similarly, patients with COPD also present symptoms associated with impaired respiratory capacity,^30^ discomfort in the legs and dysfunction of the peripheral muscles (muscle atrophy and weakness, fatigue) that limit exercise capacity.^31^, ^32^, ^33^ Given the significant muscle pain, fatigue, and weakness that patients with COVID‐19 present, it is plausible to propose a poor mechanical efficiency in COVID‐19 patients, which may, in turn affect the exercise tolerance as in patients with COPD occur.^8^

Several physiological mechanisms have been proposed to explain deficiencies in mechanical efficiency associated with the peripheral skeletal muscle. Leg discomfort and peripheral muscle dysfunction could affect exercise tolerance by alter muscle energy production during exercise and rest.^8^ This increase is usually associated with a rise in the proportion of Type II muscle fibres during exercise in patients with COPD.^34^, ^35^ This recruitment of Type II fibres is three to four times greater than in Type I fibres.^36^

During CPET, different metabolic moments took place from the start of exercise until the value of the RER was 1, and thus, the recruitment pattern of motor units was likely modified from Type I to Types IIa and IIb as the work rate increased and the fibres became progressively fatigued.^37^ The similar behaviour observed between patients with COPD and COVID‐19 suggests that a premature recruitment of less efficient Type II motor units occurred, leading to an increase in energy cost during skeletal muscle contraction. The decreased oxidative capacity of Type II fibres,^38^ the attenuated activity of some enzymes involved in the Krebs cycle,^39^, ^40^ and the greater energy cost of muscle contraction induced an unusual ATP consumption and, consequently, a reduction in DE in these patients,^8^ contributing to the early onset of muscle fatigue. Inefficiency in humans is associated with a decrease in mitochondrial efficiency.^41^ Probably, these biochemical and physiological mechanisms were, at least partly, a key factor to detect a reduced DE in COVID‐19 patients. In this study, we did not analyse the increase in energy cost during muscle contraction and its association with the recruitment of motor units, and therefore, our arguments were based on the findings of others.^8^, ^34^, ^35^, ^36^, ^37^ Unfortunately, the absence of muscle biopsies is a methodological limitation for this study. More research is warranted to clarify these arguments in patients with COVID‐19 disease.

As expected, functional capacity was significantly reduced in patients with diseases compared with the control group. Similar values of VO_2_peak have been observed in other studies in patients with COPD (12.8 mL·kg^−1^·min^−1^,^42^ heart diseases (16–18 mL·kg^−1^·min^−1^)^43^ and COVID‐19 (17.2 mL·kg^−1^·min^−1^).^44^ Interestingly, Carvalho‐Jr *et al*. found that patients with COPD who had lower FEV1 had lower VO_2_peak.^42^ They concluded that FEV1 was a predictor of VO_2_peak to determine risk and severity in patients with COPD. In our study, patients with COVID‐19 presented a VO_2_peak similar to that of patients with COPD and IHD, however, FEV1 was much higher in patients with COVID‐19 and IHD compared with patients with COPD. The ventilatory response at rest (spirometry) in patients with COVID‐19 and IHD was similar to that of the control group and higher compared with those in patients with COPD; however, cardiorespiratory response was impaired in all experimental groups with diseases during CPET. Perhaps, FEV1 is not a differential factor to predict VO_2_peak determining risk and severity in COVID‐19 patients.

No differences were detected in VO_2peak_ between the pathologies while, conversely, variances were found in the power output developed between the patients with respiratory diseases (COVID‐19 and COPD) and IHD. Under these premises, patients with IHD should probably have a higher DE (Δ*W*_ext_/ΔVO_2_) due to increased power output compared with patients with COVID‐19 and COPD. However, DE remained unchanged among the experimental groups with diseases, which represents a similar proportional raise in the increase in power output and in VO_2_ from the end of the first workload until where the respiratory exchange ratio was 1. What seems evident is that patients with COVID‐19 and COPD develop less force when pedalling during incremental CPET.

To conclude this discussion, we would like to emphasize the relationship between VO_2_peak and peak power and DE. Several studies have demonstrated an inverse correlation between DE, gross efficiency, and VO_2_max in world‐class cyclists.^19^, ^45^ The participants with the highest DE and gross efficiency had the lowest VO_2_max. The authors concluded that a low VO_2_max could be offset by increased muscle efficiency. This could be due to a physiological adaptation to training that would allow world‐class cyclists to continue at a highly competitive level. However, we found a positive correlation between DE and VO_2_peak and peak power in the experimental groups. The participants with the highest DE had the highest VO_2_peak and peak power. The participants in this study suffered from various diseases (except the control group) and were not trained. This probably suggests that the cardiorespiratory fitness and muscular fitness of the lower extremities are a determining factor for improving mechanical efficiency. The physical condition of COVID‐19 patients could be a key factor in achieving a faster recovery. It would be interesting to propose a rehabilitation programme to know the evolution of cardiorespiratory and muscular fitness and mechanical efficiency in COVID‐19 patients.

## Conclusions

Patients with COVID‐19 infection showed marked mechanical inefficiency similar to that observed in patients with COPD and IHD. The limiting factor was the muscle power developed during pedalling, which showed muscle dysfunction in patients with COVID‐19 as a determining symptomatic factor. Strength development programmes should be considered during the early phase of rehabilitation.

## Funding

None.

## Conflict of interest

All authors declare no competing interests.

## Ethical guidelines statement

The Ethics and Research Committee of the Mataró Hospital approved the study (Codi CEIm: 89/20). The protocol was conducted in accordance with the principles of the Declaration of Helsinki, Good Clinical Practice, and the applicable and local regulatory requirements. The authors of this manuscript certify that they comply with the ethical guidelines for authorship and publishing in the *Journal of Cachexia, Sarcopenia and Muscle*.^46^
